# Cytokine profiles in the aqueous humor and serum of patients with dry and treated wet age-related macular degeneration

**DOI:** 10.1371/journal.pone.0203337

**Published:** 2018-08-29

**Authors:** Jan Spindler, Souska Zandi, Isabel B. Pfister, Christin Gerhardt, Justus G. Garweg

**Affiliations:** 1 Swiss Eye Institute and Berner Augenklinik am Lindenhofspital, Bern, Switzerland; 2 University of Bern, Bern, Switzerland; University of Tennessee Health Science Center, UNITED STATES

## Abstract

**Purpose:**

To identify disease-specific cytokine profile differences in the aqueous humor (AH) (other than the vascular endothelial growth factor) between patients with dry and treated wet age-related macular degeneration (AMD) and healthy controls.

**Methods:**

This retrospective study drew on a case-series of patients diagnosed with dry AMD (*n* = 25) and treated wet AMD (*n* = 19), as well as on healthy controls (no systemic therapy; *n* = 20) undergoing phacoemulsification or vitrectomy. Samples of AH and serum were collected in parallel at the beginning of surgery. The levels of 43 cytokines were simultaneously determined using the Bio-Plex® multiplex beads system. Differences between the three groups were statistically compared using the Kruskal-Wallis H-Test after applying the Bonferroni correction for multiple comparisons (*p*<0.0012).

**Results:**

The concentrations of three cytokines were elevated in the AH of patients with dry AMD (CXCL6; *p* = 0.00067) and treated wet AMD (CXCL5, CXCL6, MIG/XCXL; all *p*<0.001) relative to those in the healthy controls. No other differences between the three groups were identified. The AH levels of seven cytokines (16%), including CXCL6, ranged below the lower limit of quantitation of the assay. Without the correction for multiple comparisons (*p*<0.05), the levels of 31 of the 43 cytokines in the AH of patients with AMD would have differed significantly from those in the control. The systemic cytokine profiles (serum) were similar in all three groups.

**Conclusions:**

No systematic differences in the AH cytokine environment were identified between patients with dry AMD and those with treated wet AMD. This finding might indicate that AMD is either the result of a persistent imbalance in the physiological tissue milieu, or that the localized process induces no significant change in the cytokine environment of the anterior ocular segment.

## Introduction

Age-related macular degeneration (AMD), particularly advanced stages of the disease, such as choroidal neovascularization (CNV) and geographic atrophy (GA), is the most common cause of irreversible vision loss in elderly individuals [1, 2, 3].

In clinical terms, the distinction between “wet” and “dry” forms of the disease is based upon the manifestation of either CNV or atrophy of the retinal pigment epithelium (RPE), the choriocapillaris and the overlying photoreceptors, respectively [4, 5]. Despite its convenience, this dichotomization does not reflect the continuum of the underlying progressive pathology of the macula with advanced age. Whereas wet AMD can be impeded (to a certain degree) by intravitreal treatment with the anti-vascular endothelial growth factor (anti-VEGF), the dry AMD component of the disease cannot be therapeutically influenced which indeed seems to be somewhat clinically distinct [1, 4, 6, 7]. However, the underlying reason for the switch towards either the atrophic, dry or the neovascular, wet form of the disease is not fully understood [8]. Since wet AMD is typically preceded by more or less prominent changes that are attributed to dry AMD, its presence could be considered as a risk factor [7]. Furthermore, advanced manifestations of both dry and wet AMD may coexist in the same eye, which argues in favor of a continuous rather than a dichotomatous process [7, 9]. Correspondingly, there exist significant overlaps in the mechanisms that underlie these seemingly disparate clinical conditions [4, 10], which is not a surprising finding for such a multifactorial disease [2] that is influenced by aging, oxidative stress, mitochondrial dysfunction, environmental factors and chronic, age-related low-grade inflammation [1]. Close correlations between AMD and various immunological/inflammatory gene polymorphisms have been reported, thereby suggesting the involvement of immune mediated processes (e.g., complement activation) and inflammation [2]. Changes in the cytokine and chemokine concentrations at both the local and the systemic levels, predominantly in patients with wet AMD, have been also documented [1, 11–17]. These cytokines appear to play an integral role in the initiation, perpetuation or subsequent down-regulation of the immune response, eventually leading to wound healing by the formation of a fibrotic scar [18, 19]. Correspondingly, histological investigations in eyes with early AMD (e.g., drusen) have revealed chronic inflammation at the RPE/choroidal interface [20]. Furthermore immunocompetent cells, such as lymphocytes and macrophages [3, 21, 22], have been observed in chorioretinal tissue that had been derived from eyes with wet AMD [2]. Generally, the molecular mechanisms that underline the development and progression of CNV, the hallmark of wet AMD, are better understood than those that are involved in the evolution of the dry form of the disease [23, 24].

It has been postulated that in the aging eye, the dysregulation of reparative (para-)inflammatory mechanisms, particularly the down-regulation of pro-inflammatory cytokines and the up-regulation of anti-inflammatory cytokines by the RPE [e.g., in response to stimulation by the deposition of advanced glycation end products (AGEs)] [25], might induce and perpetuate the low-grade chronic inflammatory process that contributes to the progression of AMD [1]. However, whether these factors are the cause or the effect of the low-grade inflammation that is associated with the progression of AMD remains to be determined [2, 6, 26].

In an attempt to understand the pathological process, changes in many different cytokines compared to healthy controls have been reported [6, 27]. Since the role of the abundancy of a single agent in the pathogenesis of AMD is difficult to estimate, we monitored and compared cytokine environmental changes by a maximally broad panel of 43 inflammatory and pro-fibrotic biomarkers in the aqueous humor (AH) and sera of patients with dry and treated wet AMD and in healthy controls. By implementing the Bonferroni correction for multiple comparisons, we endeavored to identify the most relevant beyond all significant intergroup changes in this context.

## Patients and methods

### Patients

This retrospective case series included patients with either dry or treated wet AMD or healthy controls without any relevant systemic or ocular disease (apart from senile cataract or macular hole (MH)), who were scheduled for phacoemulsification surgery and/or vitrectomy. Clinical data regarding ophthalmologic and systemic diagnoses and findings, systemic and local medications, as well as duration of the ocular symptoms (e.g., visual distortion, if manifested) were collected. For the purpose of this study, the preoperative Snellen’s best-corrected visual acuity (BCVA) was converted into Early Treatment Diabetic Retinopathy Study (ETDRS)-letter scores (with 85 letters representing a BCVA of 1.0). Samples of blood serum and AH were collected at the beginning of ocular surgery at the Berner Augenklinik am Lindenhofspital, between August 2013 and January 2016. The grading of macular changes was based upon clinical findings and OCT diagnostics in dependence on the Clinical Age-Related Maculopathy Staging System (CARMS) [28]. The following stages were distinguished: healthy controls (no chorioretinal changes), dry AMD (≥15 intermediate drusen or any large drusen, no intra- or sub-retinal fluid or hemorrhages), and treated wet AMD (signs of exudative AMD, such as serous retinal detachments, non-drusenoid RPE detachments, CNV with sub-RPE or subretinal exudations or fibrosis prior to the onset of anti-VEGF therapy, or the presence of scars consistent with AMD-treatment) [28].

Exclusion criteria included a history of systemic malignant, vascular or inflammatory co-morbidities, namely, diabetes mellitus or rheumatic diseases; a history of any previous intraocular surgery or ocular trauma in the affected eye or of intraocular inflammation; the presence or history of vitreal/(sub-)retinal hemorrhage; any ocular vascular occlusive disease; or myopia of more than 6 diopters.

The informed and written consent of all individuals concerned was obtained, in strict accordance with the tenets of the Declaration of Helsinki. The present study was approved by the local Ethics Commission of the University of Bern in Switzerland (reference number: 152/08).

### Collection of aqueous humor

Samples of aqueous humor were collected at the onset of phacoemulsification surgery. About 150 to 200 microliters of undiluted aqueous humor was obtained via aspiration through a 30-gauge needle. The samples were stored within 4 hours at -20°C for maximally 2 months and thereafter at -80°C until the time of the analysis.

### Cytokine analysis

Within four hours of collection, the aliquots of AH and serum were frozen at -20°C and stored at this temperature for up to two months, thereafter at -80°C until the time of analysis, which was conducted simultaneously for all samples.

The Bio-Plex® multiplex immunoassay beads system (Bio-Plex 100 array reader and Bio-Plex Manager software, version 6.1, Bio-Rad, Hercules, CA, USA) was used to simultaneously quantify the concentrations of 43 cytokines and chemokines according to the manufacturer instructions, as previously described [18]. A concentration standard was run in parallel on each test plate. It represented the average of triplicate standard dilutions of each corresponding chemokine/cytokine. A standard curve was generated and the sample concentrations were determined by curve-fitting. The assays were performed in a blinded manner by an experienced technician [29].

### Statistical analysis

Quantitative data are presented as mean values together with the standard deviation (SD). According to the standard curve, the lower limit of quantitation (LLOQ) of the assay working range was typically about 1 pg/ml (http://www.biorad.com). The concentrations of several cytokines ranged below the curve fit of the standards (out of range). To avoid a bias that would have been introduced by excluding these values, they were set at half of the lowest quantified level for the particular cytokine in question. Outliers were identified by a box-plot analysis (box-whisker plot). Extreme outliers (viz., values that lay 3 box-lengths beyond the box-edges) were excluded from the statistical analysis.

To ascertain whether or not the data were normally distributed the Shapiro-Wilk test was applied. Since the data did not meet the criteria of a normal distribution, the non-parametric Kruskal-Wallis H-Test was applied for the intergroup comparisons, using the level of statistical significance of *p*≤0.05. To counteract the Type I error that was attributable to the multiple comparisons, the Bonferroni correction was implemented to the level of significance, which resulted in a critical value for significance of *p*<0.0012 [30]. The statistical analyses were performed using the open source software R (Version 3.3.2–2016 RStudio, Inc.; psych package) and SPSS (version 23.0; IBM SPSS Statistics, Armonk, NY, USA) [18, 29].

## Results

### Patients

The analysis included 64 eyes from 64 patients. They were allocated to one of three groups: healthy controls (*n* = 20); dry AMD (*n* = 25); treated wet AMD (*n* = 19; Table 1). The patients with dry AMD and those with treated wet AMD were of similar age (*p*>0.05), whereas healthy controls were younger (*p* = 0.0005). The proportion of females was higher than that of males in each group (63.2% to 76.0%; chi-square test: *p* = 0.65).

**Table 1:** Demographics: Patient characteristics and clinical data for the corresponding groups.

Baseline characteristics	Healthy controls		Dry AMD		Treated wet AMD	
**Number of participants, *n* (%)**	20	(31.2)	25	(39.1)	19	(29.7)
**Number of females, *n* (%)**	14	(70.0)	19	(76.0)	12	(63.2)
**Age at sample collection, *y* mean (SD)**	74.7	(5.6)	83.5	(6.9)	84.9	(5.1)
**Best-corrected visual acuity, ETDRS-letter score, mean (SD)**	68.2	(11.2)	61.8	(19.7)	46.9	(22.0)
**Central retinal thickness, μm, mean (SD)**	237.8	(30.3)	226.2	(46.3)	236.5	(63.9)
**Choroidal thickness, μm, mean (SD)**	172.5	(59.3)	142.4	(56.7)	147.8	(61.2)

The BCVAs of the healthy controls and of the patients with dry AMD differed from those of the individuals with treated wet AMD (*p* = 0.002 and *p* = 0.02, respectively). The central retinal thickness (CRT) and choroidal thickness were similar in all groups (*p*>0.10 for all comparisons).

In patients with treated wet AMD, the mean time that elapsed between the last anti-VEGF injection and the collection of the samples was 14.9 ± 20.9 months. No differences in the cytokine levels were observed between patients who had received the last anti-VEGF injection within 6 months prior of the collection of the samples and those who had received the injection heretofore.

### Cytokine analysis

The concentrations of the different cytokines in the AH (pg/ml) spanned a broad range (Tables 2 and 3).

**Table 2:** Mean concentrations (pg/ml) and standard deviations (SD) of the 43 monitored cytokines in the aqueous humor of healthy controls and of patients with either dry or treated wet age-related macular degeneration (AMD).

Cytokine	Healthy controls		Dry AMD		Treated wet AMD		Assay Working Ranges	
	Mean (pg/ml)	SD	Mean (pg/ml)	SD	Mean (pg/ml)	SD	LLOQ (pg/ml)	ULOQ (pg/ml)
**CCL21**	976.6	931.2	1’617.3	1’100.1	2’014.8	1’627.1	21.9	3’923
**CXCL13**	0.6	1.9	0.3	0.5	0.9	1.8	0.7	1’200
**CCL27**	0.3	1.3	0.4	1.3	1.9	3.4	1.2	5’000
**CXCL5**	47.0	74.6	3’419.0	14’656.2	635.9	2’162.5	7.3	120’000
**CCL11**	5.8	4.4	8.9	8.9	8.2	3.9	1.5	3’859
**CCL24**	11.8	11.4	42.3	93.8	18.4	9.9	6.2	4’073
**CCL26**	4.1	4.6	7.7	6.7	10.9	10.2	0.9	12’109
**CX3CL1**	33.8	20.7	51.7	38.2	55.5	34.9	4.0	11’463
**CXCL6**	0.1	0.0	0.3	0.3	0.7	1.2	0.8	11’135
**GM-CSF**	49.2	25.9	93.7	87.1	67.8	33.7	5.3	35’000
**CXCL1**	28.4	21.1	45.8	35.7	48.3	16.5	3.1	7’024
**CXCL2**	1.8	1.0	3.1	2.9	3.7	2.5	4.6	13’257
**CCL1**	9.4	7.7	16.6	8.8	19.1	10.9	1.8	1’015
**IFN-γ**	3.0	3.4	7.7	7.7	8.7	7.3	2.3	20’236
**IL-1β**	0.5	0.4	0.8	0.6	1.1	0.7	0.4	7’000
**IL-2**	0.9	1.1	1.1	1.2	1.3	0.9	0.8	13’000
**IL-4**	0.6	1.4	15.4	58.7	6.3	16.2	1.2	4’804
**IL-6**	4.7	7.1	3.4	1.8	4.9	3.8	0.7	12’000
**IL-8/CXCL8**	3.6	2.9	4.5	2.5	6.1	3.0	0.5	7’640
**IL-10**	2.3	3.9	4.6	7.2	6.2	6.8	1.3	18’708
**IL-16**	4.3	5.7	9.2	9.8	14.3	10.7	2.1	34’000
**CXCL10**	41.0	40.9	58.9	72.2	122.0	112.2	1.6	7’714
**CXCL11**	1.3	2.0	2.6	2.7	4.5	4.6	0.1	2’298
**CCL2**	300.0	102.6	351.0	336.9	340.9	87.3	0.3	4’812
**CCL8**	2.3	2.2	4.5	4.7	5.7	3.5	0.3	4’056
**CCL7**	3.1	4.6	7.7	13.4	12.5	15.0	1.9	20’133
**CCL13**	0.5	0.4	1.8	3.6	1.2	0.8	0.2	3’368
**CCL22**	6.8	4.6	12.3	13.2	13.4	5.9	0.9	14’649
**MIF**	50’207.1	96’380.3	128’907.2	363’701.7	112’391.1	116’068.9	23.1	377’721
**MIG/CXCL9**	7.5	12.3	32.1	59.2	81.0	119.5	1.8	19’600
**CCL3**	0.8	0.7	1.3	0.9	1.6	1.1	0.4	1’543
**CCL15**	296.3	182.7	409.9	358.1	554.0	396.6	1.7	9’100
**CCL20**	2.9	3.1	3.2	4.4	4.6	5.7	0.3	4’675
**CCL19**	2.4	2.6	5.7	10.9	5.2	5.1	3.0	48’494
**CCL23**	5.8	5.1	13.0	12.0	13.5	10.2	1.0	14’450
**CXCL16**	490.7	198.4	459.1	266.2	539.9	221.7	0.5	2’867
**CXCL12**	82.0	60.1	107.9	107.9	162.6	148.0	8.3	115’730
**CCL17**	0.2	0.3	1.4	5.2	0.4	1.3	1.7	430
**CCL25**	117.2	153.3	243.5	444.7	260.2	196.6	20.6	114’493
**TNF-α**	5.0	3.3	9.5	7.0	10.6	6.5	0.9	13’879
**TGF-β1**	80.6	132.4	129.1	215.8	165.5	135.4	1.7	27’616
**TGF-β2**	1’288.5	738.0	1’761.9	450.0	1’742.4	831.7	14.7	30’080
**TGF-β3**	6.0	11.9	9.7	14.2	7.5	8.5	2.8	15’031

LLOQ: Lower limit of quantitation; ULOQ: Upper limit of quantitation

**Table 3:** P-values appertaining to the 43 monitored cytokines in the aqueous humor of healthy controls and of patients with either dry or treated wet age-related macular degeneration (AMD).

Cytokine	Kruskal-Wallis H-Test	Kruskal-Wallis H-Test	Kruskal-Wallis H-Test
	Healthy controls vs.	Healthy controls vs	Dry AMD vs.
	Dry AMD	Treated wet AMD	Treated wet AMD
**CCL21**	*p* = 0.034	*p* = 0.034	*p* = 0.69
**CXCL13**	*p* = 0.1546	*p* = 0.0082	*p* = 0.0113
**CCL27**	*p* = 0.075	*p* = 0.029	*p* = 0.118
**CXCL5**	*p* = 0.00265	***p* = 0.00099**	*p* = 0.26924
**CCL11**	*p* = 0.13	*p* = 0.11	*p* = 0.57
**CCL24**	*p* = 0.0035	*p* = 0.0609	*p* = 0.0609
**CCL26**	*p* = 0.0079	*p* = 0.0079	*p* = 0.2915
**CX3CL1**	*p* = 0.041	*p* = 0.041	*p* = 0.67
**CXCL6**	***p* = 0.00067**	***p* = 0.00067**	*p* = 0.10159
**GM-CSF**	*p* = 0.0014	*p* = 0.0866	*p* = 0.1096
**CXCL1**	*p* = 0.084	*p* = 0.015	*p* = 0.245
**CXCL2**	*p* = 0.0036	*p* = 0.0025	*p* = 0.0812
**CCL1**	*p* = 0.0093	*p* = 0.0093	*p* = 0.5295
**IFN-γ**	*p* = 0.02	*p* = 0.019	*p* = 0.529
**IL-1β**	*p* = 0.059	*p* = 0.008	*p* = 0.126
**IL-2**	*p* = 0.533	*p* = 0.067	*p* = 0.261
**IL-4**	*p* = 0.0091	*p* = 0.0093	*p* = 0.9518
**IL-6**	*p* = 0.39	*p* = 0.31	*p* = 0.68
**IL-8/CXCL8**	*p* = 0.082	*p* = 0.019	*p* = 0.082
**IL-10**	*p* = 0.086	*p* = 0.016	*p* = 0.129
**IL-16**	*p* = 0.0829	*p* = 0.0023	*p* = 0.0773
**CXCL10**	*p* = 0.784	*p* = 0.0078	*p* = 0.022
**CXCL11**	*p* = 0.0127	*p* = 0.0031	*p* = 0.169
**CCL2**	*p* = 0.66	*p* = 0.17	*p* = 0.1
**CCL8**	*p* = 0.0424	*p* = 0.0026	*p* = 0.0698
**CCL7**	*p* = 0.012	*p* = 0.002	*p* = 0.135
**CCL13**	*p* = 0.0048	*p* = 0.0014	*p* = 0.5613
**CCL22**	*p* = 0.0607	*p* = 0.0028	*p* = 0.0773
**MIF**	*p* = 0.222	*p* = 0.023	*p* = 0.222
**MIG/CXCL9**	*p* = 0.06656	***p* = 0.00019**	*p* = 0.02358
**CCL3**	*p* = 0.0218	*p* = 0.0048	*p* = 0.1441
**CCL15**	*p* = 0.081	*p* = 0.023	*p* = 0.081
**CCL20**	*p* = 0.91	*p* = 0.65	*p* = 0.65
**CCL19**	*p* = 0.72	*p* = 0.61	*p* = 0.61
**CCL23**	*p* = 0.014	*p* = 0.0059	*p* = 0.4767
**CXCL16**	*p* = 0.38	*p* = 0.56	*p* = 0.36
**CXCL12**	*p* = 0.982	*p* = 0.024	*p* = 0.056
**CCL17**	*p* = 0.2	*p* = 0.2	*p* = 0.62
**CCL25**	*p* = 0.0869	*p* = 0.0051	*p* = 0.0972
**TNF-α**	*p* = 0.0057	*p* = 0.0057	*p* = 0.5613
**TGF-β1**	*p* = 0.16	*p* = 0.04	*p* = 0.16
**TGF-β2**	*p* = 0.0037	*p* = 0.1705	*p* = 0.6544
**TGF-β3**	*p* = 0.16	*p* = 0.16	*p* = 0.81

After application of the Bonferroni correction, the AH-concentrations of most of the cytokines (*n* = 40) (in particular CCL21, CXCL13, CCL27, CCL11, CCL24, CCL26, CX3CL1, GM-CSF, CXCL1, CXCL2, CCL1, IFN-γ, IL-1β, IL-2, IL-4, IL-6, IL-8/CXCL8, IL-10, Il-16, CXCL10, CXCL11, CCL2, CCL8, CCL7, CCL13, CCL22, MIF, CCL3, CCL15, CCL20, CCL19, CCL23, CXCL16, CXCL12, CCL17, CCL25, TNF-α, TGF-β1, TGF-β2, and TGF-β3) were similar in all three groups [healthy controls, dry AMD and treated wet AMD (*p*>0.0012)].

**Fig 1:**
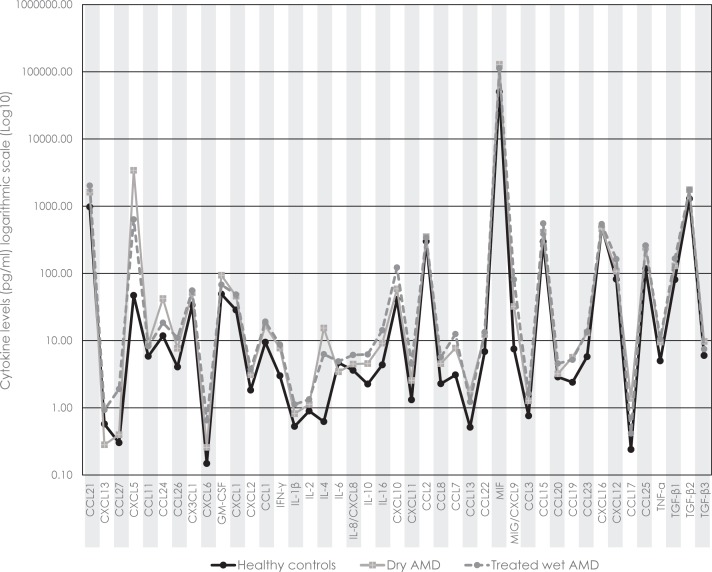
Mean concentrations of the 43 monitored cytokines in the aqueous humor of healthy controls (continuous black trace) and of patients with either dry age-related macular degeneration (AMD; continuous grey trace) or treated wet AMD (dashed grey trace), represented on a logarithmic scale. Note: The presentation of non-continuous data as a line graph permits an improved estimation of concentration changes not only for individual cytokines (points) but also for the cytokine environment as a whole.

Eyes with treated wet AMD exhibited the highest absolute AH-concentrations in 31 of the 43 analyzed cytokines (72%). In the dry AMD group, the AH-concentrations of CXCL5, CCL11, CCL24, GM-CSF, IL-4, CCL2, CCL13, MIF, CCL19, CCL17, TGF-β2, and TGF-β3 were higher than those in either the healthy controls or the eyes with treated wet AMD (Fig 1, Table 2).

**Fig 2:**
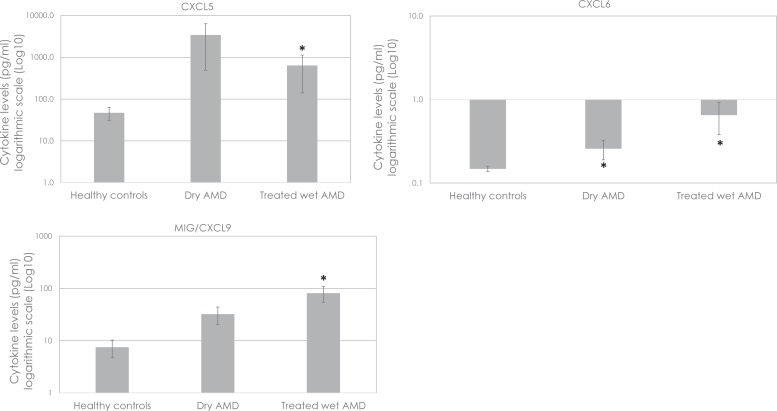
Mean concentrations (pg/ml) of the three cytokines/chemokines CXCL5, CXCL6 and MIG/CXCL9 in the aqueous humor of patients with either dry age-related macular degeneration (AMD) or treated wet AMD showing relevant differences (*, *p*<0.0012) in their levels when compared to healthy controls. Whiskers represent the standard error of the mean. Values are represented on a logarithmic scale.

The concentrations of three cytokines were 1.7 to 72.8-fold higher (*p*<0.0012) in patients with either dry AMD [CXCL6 (*p* = 0.00067)] and/or treated wet AMD [CXCL5 (*p* = 0.00099), CXCL6 (*p* = 0.00067), MIG/CXCL9 (*p* = 0.00019)] than in the healthy controls, with significant intergroup differences being registered after the application of the Bonferroni correction (Fig 2, Table 3).

No intergroup differences in the AH-concentrations of the cytokines were observed between eyes with dry AMD and those with treated wet AMD (all *p*>0.01).

CXCL6 and CCL7 were the only cytokines whose AH-concentrations increased with progression of age (*p* = 0.0006439 and *p* = 0.0006956, respectively; other cytokines: *p*>0.0012).

In no instance were any intergroup differences observed in the serum concentrations of the monitored cytokines [*p*>0.0012 (Tables 4 and 5)]. The cytokine concentrations were 1.2 to 5775.6-fold higher in the serum than in the corresponding AH samples with the following exceptions: CXCL5 (dry AMD only), GM-CSF, CCL2, TGF-β1 (healthy controls only), TGF-β2 and TGF-β3. No age-correlated changes in any of the cytokines were identified in the serum samples (*p*>0.0012).

**Table 4:** Mean concentrations (pg/ml) and standard deviations (SD) of the 43 monitored cytokines in the sera of healthy controls and of patients with either dry or treated wet age-related macular degeneration (AMD).

Cytokine	Healthy controls		Dry AMD		Treated wet AMD		Assay Working Ranges	
	Mean (pg/ml)	SD	Mean (pg/ml)	SD	Mean (pg/ml)	SD	LLOQ (pg/ml)	ULOQ (pg/ml)
**CCL21**	7’971.5	2’008.0	14’357.7	17’653.8	19’630.5	18’509.2	21.9	3’923
**CXCL13**	23.3	13.1	33.1	25.4	35.1	25.3	0.7	1’200
**CCL27**	1’612.3	428.2	2’293.4	1’188.6	2’587.4	1’177.7	1.2	5’000
**CXCL5**	724.7	274.1	1’217.4	1’155.9	1’374.2	1’039.9	7.3	120’000
**CCL11**	48.4	9.5	57.5	24.1	76.2	36.6	1.5	3’859
**CCL24**	371.6	196.3	584.0	560.2	433.0	250.9	6.2	4’073
**CCL26**	42.0	14.1	49.9	44.6	63.9	49.4	0.9	12’109
**CX3CL1**	166.2	46.7	242.0	152.0	271.7	179.3	4.0	11’463
**CXCL6**	32.1	10.1	34.6	18.4	45.8	24.1	0.8	11’135
**GM-CSF**	22.9	18.4	32.9	30.0	43.5	41.3	5.3	35’000
**CXCL1**	318.0	63.1	328.5	117.4	365.1	98.9	3.1	7’024
**CXCL2**	494.4	265.4	678.3	726.3	1’083.1	982.7	4.6	13’257
**CCL1**	66.7	10.3	75.5	40.7	87.9	45.8	1.8	1’015
**IFN-γ**	63.0	22.4	74.8	46.9	93.4	50.3	2.3	20’236
**IL-1β**	5.2	1.7	5.0	2.2	5.3	1.9	0.4	7’000
**IL-2**	10.9	4.2	11.7	6.1	13.6	5.9	0.8	13’000
**IL-4**	44.0	6.7	52.4	25.7	56.9	28.7	1.2	4’804
**IL-6**	6.5	2.9	8.8	3.7	10.3	4.1	0.7	12’000
**IL-8/CXCL8**	10.9	7.0	20.1	27.2	37.0	45.4	0.5	7’640
**IL-10**	70.8	25.5	88.1	63.4	105.5	79.0	1.3	18’708
**IL-16**	285.1	73.9	458.9	246.4	714.5	589.4	2.1	34’000
**CXCL10**	191.5	116.2	483.0	1124.9	313.1	203.7	1.6	7’714
**CXCL11**	38.8	9.1	108.0	189.1	84.6	65.0	0.1	2’298
**CCL2**	60.1	14.7	81.6	51.6	95.4	56.2	0.3	4’812
**CCL8**	88.7	21.6	151.7	143.5	160.1	131.0	0.3	4’056
**CCL7**	128.3	44.5	154.6	109.4	184.0	117.8	1.9	20’133
**CCL13**	81.8	24.8	115.4	80.2	125.3	78.0	0.2	3’368
**CCL22**	1010.5	1063.4	641.5	330.4	1022.7	598.4	0.9	14’649
**MIF**	68’484.2	78’856.9	166’888.4	259’605.2	254’498.5	239’185.4	23.1	377’721
**MIG/CXCL9**	432.0	228.1	2’200.1	6’308.5	1’221.1	1’222.3	1.8	19’600
**CCL3**	8.1	2.7	9.9	5.9	11.2	3.6	0.4	1’543
**CCL15**	5’487.2	2’218.7	9’693.6	6’064.3	10’720.8	7’705.4	1.7	9’100
**CCL20**	18.0	12.0	19.0	13.1	18.8	9.3	0.3	4’675
**CCL19**	512.1	344.9	679.1	648.3	802.2	857.2	3.0	48’494
**CCL23**	350.4	116.7	399.1	192.6	427.0	226.1	1.0	14’450
**CXCL16**	580.9	155.1	689.7	277.5	857.2	394.3	0.5	2’867
**CXCL12**	929.8	298.2	1172.3	774.1	1’598.1	1’319.1	8.3	115’730
**CCL17**	206.9	160.7	275.3	283.3	344.4	421.4	1.7	430
**CCL25**	810.8	286.0	1’063.3	713.7	1’531.1	913.4	20.6	114’493
**TNF-α**	53.4	11.2	72.6	53.4	87.7	61.5	0.9	13’879
**TGF-β1**	13.6	0.0	205.2	376.9	273.0	548.5	1.7	27’616
**TGF-β2**	3.1	0.0	107.6	423.6	3.1	0.0	14.7	30’080
**TGF-β3**	1.5	1.9	4.6	7.1	5.0	9.2	2.8	15’031

LLOQ: Lower limit of quantitation; ULOQ: Upper limit of quantitation

**Table 5:** P-values appertaining to the 43 monitored cytokines in the sera of healthy controls and of patients with either dry or treated wet age-related macular degeneration (AMD).

Cytokine	Kruskal-Wallis H-Test	Kruskal-Wallis H-Test	Kruskal-Wallis H-Test
	Healthy controls vs.	Healthy controls vs	Dry AMD vs.
	Dry AMD	Treated wet AMD	Treated wet AMD
**CCL21**	*p* = 0.6	*p* = 0.6	*p* = 0.65
**CXCL13**	*p* = 0.36	*p* = 0.36	*p* = 0.99
**CCL27**	*p* = 0.17	*p* = 0.12	*p* = 0.51
**CXCL5**	*p* = 0.45	*p* = 0.45	*p* = 0.45
**CCL11**	*p* = 0.27	*p* = 0.27	*p* = 0.27
**CCL24**	*p* = 0.7	*p* = 0.7	*p* = 0.7
**CCL26**	*p* = 0.77	*p* = 0.77	*p* = 0.77
**CX3CL1**	*p* = 0.2	*p* = 0.2	*p* = 0.85
**CXCL6**	*p* = 1.0	*p* = 0.27	*p* = 0.27
**GM-CSF**	*p* = 0.64	*p* = 0.64	*p* = 0.64
**CXCL1**	*p* = 0.69	*p* = 0.54	*p* = 0.54
**CXCL2**	*p* = 1.0	*p* = 0.6	*p* = 0.6
**CCL1**	*p* = 0.74	*p* = 0.74	*p* = 0.74
**IFN-γ**	*p* = 0.54	*p* = 0.28	*p* = 0.36
**IL-1β**	*p* = 0.9	*p* = 0.9	*p* = 0.9
**IL-2**	*p* = 0.77	*p* = 0.59	*p* = 0.61
**IL-4**	*p* = 0.5	*p* = 0.5	*p* = 0.71
**IL-6**	*p* = 0.174	*p* = 0.076	*p* = 0.259
**IL-8/CXCL8**	*p* = 0.29	*p* = 0.12	*p* = 0.12
**IL-10**	*p* = 0.74	*p* = 0.74	*p* = 0.74
**IL-16**	*p* = 0.076	*p* = 0.076	*p* = 0.381
**CXCL10**	*p* = 0.47	*p* = 0.3	*p* = 0.47
**CXCL11**	*p* = 0.27	*p* = 0.12	*p* = 0.51
**CCL2**	*p* = 0.41	*p* = 0.22	*p* = 0.41
**CCL8**	*p* = 0.27	*p* = 0.27	*p* = 0.68
**CCL7**	*p* = 0.79	*p* = 0.65	*p* = 0.65
**CCL13**	*p* = 0.58	*p* = 0.58	*p* = 0.61
**CCL22**	*p* = 0.48	*p* = 0.46	*p* = 0.05
**MIF**	*p* = 0.213	*p* = 0.057	*p* = 0.141
**MIG/CXCL9**	*p* = 0.18	*p* = 0.12	*p* = 0.72
**CCL3**	*p* = 0.4	*p* = 0.14	*p* = 0.22
**CCL15**	*p* = 0.037	*p* = 0.037	*p* = 0.745
**CCL20**	*p* = 0.88	*p* = 0.88	*p* = 0.88
**CCL19**	*p* = 0.97	*p* = 0.97	*p* = 0.97
**CCL23**	*p* = 0.87	*p* = 0.87	*p* = 0.87
**CXCL16**	*p* = 0.3	*p* = 0.14	*p* = 0.3
**CXCL12**	*p* = 0.48	*p* = 0.48	*p* = 0.48
**CCL17**	*p* = 0.97	*p* = 0.97	*p* = 0.97
**CCL25**	*p* = 0.6	*p* = 0.14	*p* = 0.16
**TNF-α**	*p* = 0.41	*p* = 0.41	*p* = 0.41
**TGF-β1**	*p* = 0.086	*p* = 0.086	*p* = 0.915
**TGF-β2**	*p* = 0.21	-	*p* = 0.21
**TGF-β3**	*p* = 0.66	*p* = 0.66	*p* = 0.96

## Discussion

Our results revealed an upregulation of CXCL5, CXCL6 and MIG/CXCL9 (*p*<0.0012) in the AH of eyes with dry and stable treated wet AMD, when compared to healthy controls. These were the only cytokines whose up-regulation remained significant after the application of the Bonferroni correction for multiple comparison. Heretofore, an additional 31 cytokines had qualified for this designation when considering a level of significance of *p*<0.05. These findings indicate that an analysis of the level of a single cytokine in clinical samples may suffer from the weakness of attempting to detect and interpret a single point change in the complex pathomechanism of AMD. Furthermore, it is challenging to estimate modulations in the local cytokine environment/milieu at the lesion site by specimens taken from the anterior ocular compartment rather than from where the pathology actually takes place. It remains a matter of speculation whether or not the AH is indeed representative of the relatively small volume of the macular RPE/Bruch's membrane complex. Likely, only the most abundant changes in the cytokine concentrations at the lesion site will be detectable in the constantly renewing AH. This circumstance may partially explain why the number of cytokines for which intergroup differences were detected was not larger than it was. The three that qualified for this distinction, namely, CXCL5, CXCL6 and MIG/CXCL9, probably figure in a true disease-associated effect. Interestingly, the differences in their concentrations were between the healthy controls and the two pathological sub-groups, *not* between the two forms of the AMD pathology. This finding and the fact that no systematic change in the cytokine environment was detected between dry AMD and treated stable wet AMD (Fig 1) suggests that a persistent imbalance in the local milieu might exist in this disease, with smooth transitions occurring between dry and wet AMD pathogenesis [4]. However, the fact that we investigated samples of treated stable wet AMD leads to the speculation that we looked at an inactive state of the disease with possible cytokine downregulation. Nevertheless, CNV reactivation is possible at any time so that it might be at least partially similar to the situation prior to the primary activation of the lesion.

Inflammatory activity at a subclinical level could indeed figure in AMD, in analogy to the situation that is observed in the pseudoexfoliation syndrome, which is another age-dependent degenerative disease with an inflammatory background. This postulate fits well with the concept of inflammaging or immunosenescence, an age-related inflammatory response to aging changes, found in many organs [31–37]. Since aging is the strongest risk factor for the development and progression of AMD, the existence of a link between the pathogenesis of the disease and local immunosenescence is very likely [2, 38].

In the ocular tissue of patients with AMD, an accumulation of T-cells has been observed. Consequently, it has been proposed that lymphocytes may play a pivotal role in the breakdown of Bruch’s membrane, in RPE-atrophy and in the onset CNV in early and late stages of AMD [39, 40]. Monokine induced by interferon-gamma (-γ) (MIG/CXCL9), which was up-regulated in treated wet AMD, is known to be a crucial chemokine in many inflammatory processes, particularly in those that are mediated by T-cells [41]. It is specific for T-cell chemotaxis and cell attraction and was found to be overexpressed in the RPE/Bruch’s membrane/choriocapillaris complex of aging mice [42]. Although our sample number may have been too small to detect age-related changes in in the concentrations of MIG/CXCL9 within the AH or the serum, Torres and coworkers recently described serum MIG/CXCL9 levels to increase with progression of age by using a large population-based cohort study [43, 44]. Shi and colleagues reported that MIG/CXCL9, interferon-γ inducible protein 10-kDa (IP-10, CXCL10), and interferon-γ inducible T-cell alpha (α) chemoattractant (I-TAC, CXCL11) are expressed in RPE [45]. However, the specific functions of these chemokines in angiogenesis remain to be determined [45–48]. As an indicator of its clinical relevance, Jonas et al. reported an association between the severity of retinal edema and elevated concentrations of MIG/CXCL9 [13]. CNV, on the other hand, does not appear to be driven primarily by inflammatory activity, since a relevant effect was not detected in AMD-patients who had undergone treatment with corticosteroids in addition to anti-VEGF therapy in a prospective randomized clinical trial [49].

The finding that elevated levels of MIG/CXCL9 were apparent only in treated wet AMD could suggests that this change is not only age-related [43, 44], but also a marker of retinal diseases with inflammatory components such as AMD [50] at a late, steady-state stage, which deserves further attention.

In the vast majority of our patients with wet AMD, an interval of more than 6 months had elapsed between the last anti-VEGF injection and the time at which the samples were collected. Hence, the influence of intravitreal anti-VEGF treatment should be negligible [51, 52].

In the context of ocular diseases, data appertaining to the neutrophil-specific pro-inflammatory and chemoattractive cytokines/chemokines CXCL5 (ENA-78) and CXCL6 (GCP-2) are sparse. Both chemokines show a high homology in primary structure (chemotactic potency: CXCL6 > CXCL5 [53]) and they both interact with CXCR-2 (CXC chemokine receptor 2) [54]. The present study is one of the first to identify noticeably elevated levels of these cytokines/chemokines in the eyes of patients with dry and/or treated wet AMD relative to the situation in the healthy controls, which remained statistically significant even after the correction for multiple comparisons. The extent to which these increases contribute to the pathogenesis and/or persistence of AMD remains to be addressed.

A similar up-regulation has been reported also for late forms of the pseudoexfoliation syndrome with luxation of the intraocular lens [29] and in eyes with epiretinal membranes [18]. Parmar et al. reported increases in the levels of several cytokines, including CXCL5, which have been identified as an acute stress response to intense light in immunodeficient mice with a disrupted visual cycle and as pivotal factors in the development of retinal degeneration [55]. An association between chronic light-induced damage and AMD has been reported, and this circumstance could account for the almost 14-fold up-regulation of CXCL5 [56]. In patients with dry AMD and in those with treated wet AMD, CXCL6 was up-regulated relative to the situation in the healthy controls. Since the AH-levels of CXCL6 (min. 0.1 pg/ml in healthy controls; max. 0.7 pg/ml in treated wet AMD) ranged below the LLOQ for the used immunoassay system (0.8 pg/ml) the detected differences may reflect not only a true disease-based change, but also partially measurement inaccuracies. Until this latter possibility can be excluded, an interpretation of this finding is not possible.

Moreover, we did not observe any difference in serum cytokine profiles neither between the three compared groups nor with progression of age within the analyzed pool of patients. This might indicate that AMD is a local ocular process without measurable systemic cytokine environmental changes which has already been discussed controversially (e.g., TNF-α) [57, 58]. However, an age-related low-grade systemic/generalized inflammation within a similar degree in healthy controls and AMD patients cannot be excluded.

Beyond the limitations of this study, the healthy controls were approximately 10 years younger than the patients with dry or treated wet AMD. A possible influence of age on the results cannot therefore be excluded. However, since a correlation between age and the AH-levels of the cytokines was detected in only two instances [CXCL6 (*p* = 0.0006) and CCL7 (*p* = 0.0007)] and not at all between age and the serum concentrations, any effect, if it existed, would be marginal. Nevertheless, in future studies, an age-matched group of healthy controls would be included to enhance the power of the intergroup differences. Given that no age-related differences between dry and treated wet AMD were identified, the absence of intergroup differences is probably a reliable finding. Finally, since a very robust level of significance was employed in the present study (Bonferroni correction), only differences of high significance (*p*<0.0012) were considered. Hence, we cannot exclude the possibility that some relevant results might have been thereby dismissed. Nevertheless, in terms of biological relevance, we believe that the application of such a high level of significance contributed to the strength of our findings.

For 40 of the 43 monitored cytokines, we could identify no intergroup differences in concentration. Our findings respecting some of these cytokines conflict with existing data, which reveal specific associations between their levels and the pathogenesis of wet AMD [1, 11, 13, 17, 25, 26, 48, 59–63] as well as aging [64–66], either locally in the AH or systemically in the blood serum. Of particular note are our findings respecting IL-4 and IL-10, which, in contrast to existing data, were not up-regulated. An up-regulation of these two cytokines would indeed be reasonable, since both appear to be involved in the pathogenesis of AMD [3, 11, 21, 22, 26, 59, 67–70] and the process of aging [22, 25, 64, 43]. The discrepancies between our own and existing data may be partially explained by the circumstance that we chose to evaluate not only individual cytokines but also the cytokine environment as a whole, which necessitated a correction for multiple comparisons. As a consequence, the concentrations of 31 of the monitored cytokines fell below the level of statistical significance (*p*>0.0012). The heterogeneity of the AMD-stages that were investigated in various published studies may also have contributed to the different outcomes [67].

In conclusion, three of the 43 monitored cytokines (7%) were up-regulated in the AH of AMD-patients relative to the situation in the healthy controls. No differences between dry and treated wet AMD were identified. Our data support existing evidence that inflammatory/immunological processes play a role in the pathogenesis of AMD. The finding that 31 of the 43 monitored cytokines (72%) were dysregulated in patients with wet AMD relative to the situation in the healthy controls at a significance level of *p*<0.05 affords strong evidence that data appertaining to the concentrations of individual cytokines are barely interpretable on this statistical basis. The circumstance that the AH-levels of seven of the 43 tested cytokines (16%) hovered below the LLOQ for the immunoassay contributes to the difficulty of interpretation. Consequently, at the present time, estimating the specific role of these cytokines in the pathogenesis of AMD is challenging, since a relevant effect at the tissue level of the RPE/Bruch’s membrane/choriocapillaris complex cannot excluded in the face of existing data.
